# A Deep-Learning Model for Underwater Position Sensing of a Wake’s Source Using Artificial Seal Whiskers

**DOI:** 10.3390/s20123522

**Published:** 2020-06-22

**Authors:** Mohamed Elshalakani, Muthukumar Muthuramalingam, Christoph Bruecker

**Affiliations:** Department of Mechanical Engineering and Aeronautics, City University of London, Northampton Square, London EC1V 0HB, UK; Muthukumar.Muthuramalingam@city.ac.uk (M.M.); Christoph.Bruecker@city.ac.uk (C.B.)

**Keywords:** biomimetics, underwater target tracking, deep learning, smart sensors, underwater robotics

## Abstract

Various marine animals possess the ability to track their preys and navigate dark aquatic environments using hydrodynamic sensing of the surrounding flow. In the present study, a deep-learning model is applied to a biomimetic sensor for underwater position detection of a wake-generating body. The sensor is composed of a bundle of spatially-distributed optical fibers that act as artificial seal-like whiskers and interact with the body’s wake in the form of time-variant (bending) deflections. Supervised learning is employed to relate the vibrations of the artificial whiskers to the position of an upstream cylinder. The labeled training data are prepared based on the processing and reduction of the recorded bending responses of the artificial whiskers while the cylinder is placed at various locations. An iterative training algorithm is performed on two neural-network models while using the 10-fold cross-validation technique. The models are able to predict the coordinates of the cylinder in the two-dimensional (2D) space with a high degree of accuracy. The current implementation of the sensor can passively sense the wake generated by the cylinder at *Re* ≃ 6000 and estimate its position with an average error smaller than the characteristic diameter *D* of the cylinder and for inter-distances (in the water tunnel) up to 25-times D.

## 1. Introduction

According to the market research report presented in [1], the size of the underwater robotics market was estimated at USD 2.52 billion in 2017, which represented about 7.6 per cent of the global robotics market size. With water covering about 71 percent of the earth’s surface, underwater robotics has allowed for a broad range of applications in military, commerce, and science. Generally, the robot’s perception of its surrounding environment is essential for performing tasks, avoiding risks, and navigation. With the help of surveillance sensors, robots can explore the unknown offshore world securely, despite the hazardous and unpredictable underwater environment. However, further progression in the development of navigation and object-tracking sensors faces a variety of technical challenges [2,3,4].

Mostly, vision and sonar systems have been used for underwater surveillance. However, both techniques feature some serious drawbacks. Firstly, vision-based sensors are known for their limited perceptible range, which confines the use of cameras to the near surrounding space. The absorption and scattering of light cause the underwater environments to be muddy and cloudy, which results in images that can hardly be featured [5,6]. Additionally, an artificial source of light energy is required in order to use a vision-based underwater sensor due to the low level of natural illumination in the deep water [7]. Similarly, the transmission of acoustic waves is required for a sonar system to estimate the location of the objects in the surrounding space [8]. Because both of the techniques perform active sensing, the location of the emitter is revealed, while a high level of stealth is often required in surveillance applications. On another hand, sound emissions have been declared to be life-threatening to many marine animals. Schrope, M. reported several death cases of beaked and minke whales due to the emissions of the sonar systems used by the US Navy [9]. Finally, the energetic efficiency of both techniques is questionable. It has been shown that the use of sonar sensing in a small autonomous-underwater-vehicle (AUV) leads to significant inflation of the total consumed power [8].

Marine life is full of examples that can be studied to mine for new techniques that can help to overcome the current challenges of underwater sensing. Fish and marine animals demonstrate the ability to use their passive mechanical sensors for object-detection and navigation under water, even with a partial or full lack of both visual and bio-sonar. The blind cavefish is able to swim at high-speeds while avoiding collisions with nearby objects. This fish developed the ability, known as hydrodynamic imaging, to blindly sense the motion of water and recognize the surrounding objects due to the usually-dark environment where it lives [10,11,12]. Pinnipeds (such as sea lions and seals) have the ability to detect water disturbances using their facial mechanoreceptors, known as whiskers. They can use the acquired information to navigate the surrounding environment, identify certain objects, and track their preys. Hanke et al. demonstrated that the hydrodynamic trail of some preys remain detectable in water for several minutes [13,14]. This gives Pinnipeds the ability to track far targets that can hardly be detected by vision. It was experimentally shown that the Harbor seal can follow the path undertaken by a submarine, even with an inter-distance of about 40 m [15]. It was also able to detect the direction of the submarine’s wake even with a 90° encountering angle. A control experiment was conducted under the same conditions after the motion of the tracking seal’s whiskers had been impeded. In this experiment, it was proven that the seal cannot detect the submarine’s wake without its whiskers. In another study, a blindfolded Harbor seal was able to follow the hydrodynamic trail generated by a pilot seal after it had left the water [16]. Harbour seals were also capable of detecting sinusoidal vibrations in water with speeds that ranged from 0.245 to 1.8 mm·s^−1^ and frequencies ranging from 10 to 100 Hz [17]. Last but not least, seals were also found to be capable of distinguishing sizes and shapes of objects by sensing their hydrodynamic wakes [18].

Various sensing techniques have been adopted from biological models and used for the characterization of underwater environments [19,20,21,22]. Such sensors may assist or even replace the sonar and vision functionalities in some applications that require certain levels of accuracy, stealth, or environmental impact. Kottapalli et al. developed a MEMS pressure sensor for AUVs that mimics the fish lateral-line [23]. Motivated by Dehnhardt’s experiments of Harbor seals, Eberhardt et al. presented a system of artificial whiskers that produced vibration signals that were related to the hydrodynamic trail of a pilot submarine [24]. We believe that further development of biomimetic sensory systems would help marine robots to expand their perception of the surrounding fluid motion.

Previous studies about seal’s sensing abilities have guided us to explore how the seal interprets the perceived whiskers’ vibrations for hydrodynamic detection. Different vortex structures were shown to carry information about the direction of the disturbance source to the seal [25,26]. Wieskotten et al. showed that the seal is able to track a propelled and gliding submarine, even with two different wake’s inner fields [27]. By fluid-whisker interaction, the seal is able to detect the direction in which it needs to swim to track the swimming body [24]. The wake of a cylinder (*Re* ∈ [4000, 6000]), which mimics the trail generated by a prey (fish), was reported to induce time-variant deflection responses of artificial seal-like whiskers [28]. With the whiskers distributed over both sides of the frontal area of the pinniped’s head, the generated wake affects each whisker differently, depending on its adjacent local flow structure. We believe that, by comparing the vibrations of different whiskers, information about the location of the wake’s source can be deduced. The whiskers’ vibrations in [28] were measured by tracking the whisker tips’ motion while using a camera. Other embedded techniques, such as the fiber-Bragg-grating [29,30] and strain gauges, can also be used to record the bending responses of such whisker beams.

Neural systems of the brain do a unique job in exploiting the perceived noisy raw data. In the visual cortex, signals that contain visual information (e.g., colours, intensities, etc.) are interpreted as recognizable faces and objects. In the auditory cortex, the noisy vibration signals (i.e., amplitudes and frequencies) are interpreted as meaningful sounds. Likewise, it is believed that the seal’s brain is capable of translating the perceived whisker vibrations into a hydrodynamic image of the surrounding aquatic environment. Accordingly, we aim to realize an artificial-neural model that relates the vibration signals of an array of artificial whiskers to the source of the disturbance that causes these vibrations. In 2017, the same approach was taken by [31] for developing a goal-driven neural-network model that mimics the rodent’s vibrissal-trigeminal system. They built a 31-whisker array that performed as a bio-physical realistic model the rodent’s vibrissal system. As the whiskers swept across various objects with different shapes, they collected the data from different sweeps (i.e., torques and forces at the base of each whisker) and used them to train the networks to perform a three-dimensional (3D) shape recognition task.

Whisker-like artificial systems have been utilized in engineering applications for both active and passive sensing. Pearson et al. demonstrated the use of active whisker-arrays for increasing the amount and quality of tactile sensory information for mobile robots inspired by the vibrissal sensory systems of small mammals, such as rats [32]. Similar whisker-like tactile systems have been developed and tested for recognizing the shapes and identities of different surrounding objects [33,34,35]. The hitherto known studies of the use of whiskers in underwater sensing have focused on finding the sensitivity limits of such structures in sensing flow disturbances in the aquatic environment. A special undulated design of the cross-section along the whisker body was found to suppress the vortex-induced vibrations of the whisker, thus removing the unwanted responses induced by the seal’s own propulsive motion [36,37]. These results inspired Alvarado et al. to propose a whisker-type sensor design that could be tuned in order to amplify certain hydrodynamic signals and suppress others (e.g., noise) [38]. Recently, experimental studies were carried out in our group for underwater sensing using arrays of whiskers, where it was proven that the deflection signals of artificial whiskers can decode the specific shedding frequency of a Kármán vortex street [28]. The studies also highlighted the importance of the vortex-induced pressure-gradients as a major contribution to the jerky response of the whiskers. These studies have established the basis for the present work.

### Objective

While previous studies have proposed and developed promising underwater sensors based on seal whiskers’ behavior, the vast majority could only identify the hydrodynamic wakes generated by different objects and some of their characteristics. Up to now, to the best of the authors’ knowledge, whisker-like sensors were not used for the position detection of a wake’s source or for navigation applications in autonomous and non-autonomous underwater vehicles. In this study, we aim to employ the ability of seal whiskers to detect surrounding water movements in the development of an underwater sensor that detects the position of the source of an upstream flow disturbance. Using supervised learning, we develop two models that relate the whiskers’ vibrations, on both sides of a pinniped’s head model, to the 2D coordinates of the position of an upstream wake-generating body. The proposed sensory system implements a stealthy and life-like way of hydrodynamic sensing. It is meant to build the basis for the development of a replacement or complementary device to the current conventional underwater tracking systems.

## 2. Materials and Methods

### 2.1. Physical Model

**Figure 1:**
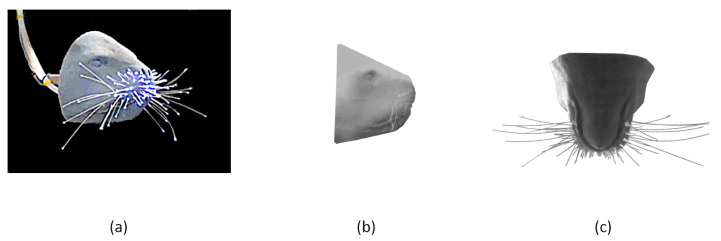
Three-dimensional (3D) printed model of the pinniped head. (**a**) captured image of the head while the optical fibres are illuminated. (**b**) side view (**c**) plan view of the head.

A copy of a sea lion’s head was designed and 3D printed at City, University of London based on the CT scan data of a real sea lion that was acquired from Museo Delle Scienze, Italy. Optical fibers were gathered and illuminated from one end and inserted through holes from the back side of a pinniped’s head model. The fibers’ free endings, which are existing out of the holes from the front side of the head model, perform as artificial whisker-like transducers. The diameter, lengths, and locations of the fibers were selected so that their dimensions and spatial distribution are comparable to those of real whiskers based on the analysis presented in [39,40]. The fibers are made of polymethylmethacrylate (PMMA), which has a Young’s Modulus of about 3.5 GPa [41], which is similar to the real whisker’s [42]. An illumination source was attached to the end of the fibers that act as a guide to the light until it exists from their tips. Figure 1 shows the 3D printed head and the attached fibre cables that act as artificial whiskers. All of the fibers have the same diameter of 0.75 mm that is constant over their lengths. We capture the motion of 12 artificial whiskers that have the same length and were previously shown in [28] to have a similar response to real whiskers.

### 2.2. Experiment

**Figure 2:**
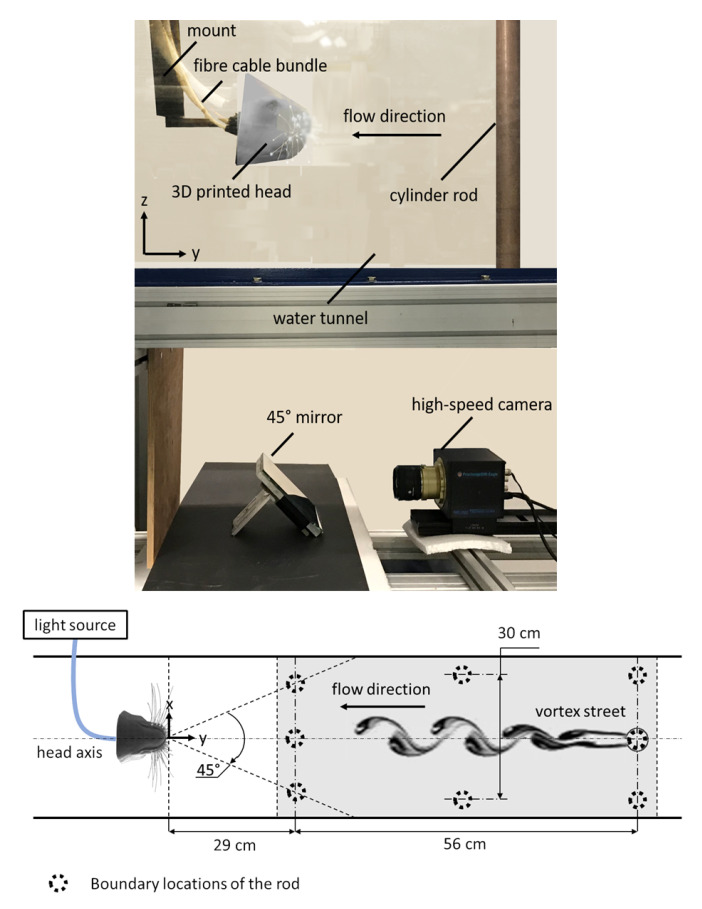
Experimental set-up of the artificial head and the flow disturbance inside the water tunnel. The side view (**top**) shows the optical setup of a high-speed camera underneath the test section and a 45° mirror that are used to monitor the motion of the illuminated whisker tips. The plan view (**bottom**) shows the boundary locations of the cylinder during different tests. The recorded data of the whiskers’ vibrations due to the existence of the cylinder at different locations are used for training the learning models. The origin of the coordinate system is placed at the intersection of the head axis with its frontal face.

Figure 2 shows the experimental setup used for generating the data, which will then be processed to prepare the training datasets (input-target pairs) of the learning models. The head model is placed and fixed in the center of an open-surface water tunnel with a transparent test section (120 cm × 50 cm × 40 cm: length × width × depth), which processes a water flow of 30 cm/s free-stream velocity. A 35 mm cylindrical metal rod is vertically placed in the open-surface flow in the upstream direction of the head. A high-speed camera records the coordinates of 12 whiskers’ tips that are distributed on both sides of the head and subjected to the hydrodynamic trail of the cylindrical rod which can be located at different locations in the water tunnel.

The wake generated by the cylinder can be characterized by the Reynolds and Strouhal numbers; *Re* and *St*, that are given by Equations (1) and (2):

(1) $$St=\frac{fD}{U_{\infty }}$$

(2) $$Re=\frac{U_{\infty }D}{\nu }$$

where *f* is the Strouhal frequency, *D* is the diameter of the rod, *U*_∞_ is the free-stream flow velocity, and ν is the kinematic viscosity of water. For cylindrical bluff bodies and the present configuration; *Re* ≃ 6000 and *St* ≃ 0.2 [43], a repeating pattern of vortices (Kármán vortex street), is formed in the wake of the cylinder, which interacts with the artificial whiskers, which act like cantilever beams, in the form of bending deflections.

### 2.3. Data Acquisition

**Figure 3:**
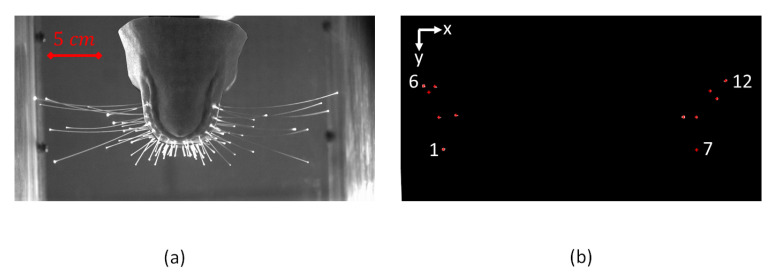
Camera views of the two recording modes: (**a**) grey-level compressed mode. (**b**) binary mode with centroid calculation. The grey-level image is masked before binarization so that only the 12 whiskers’ tips of interest are visible. The whiskers are numbered from 1 to 12 such that whiskers no. 1, 6, 7 and 12 indicate the front-left, rear-left, front-right and rear-right whiskers respectively.

The high-speed camera (ProcImage 500-Eagle high-speed camera, 1280 px × 1024 px, Photon Lines Ltd, Bloxham, UK) is used to record the bottom view of the head and the illuminated whisker tips at a frame rate of 200 fps. Figure 3 shows the image captured by the camera in two different modes: (1) eight-bit grey level mode. (2) binary mode with centroid detection of white connected pixels in a selected area in the frame. The 12 coordinates of the centroids of the white dots that correspond to the whiskers’ tips are extracted from the second-mode frames and then saved for further processing. The position trajectories of the tips of the outer whiskers that possess the largest length (l≃10 cm) are recorded throughout various tests. The inner whiskers are less sensitive to the disturbance generated by the upstream rod that is placed at different locations relative to the head due to their lengths and orientations. For each of 32 different positions of the cylinder in the two-dimensional (2D) domain, a separate recording of the image-coordinates of the whisker tips (that has a period of approximately one minute) is saved for further processing. For the present set-up, the recording has to be stopped each time before the position of the cylinder is changed.

### 2.4. Data Processing and Feature Extraction

Using the recorded tip coordinates in the successive camera frames, the wake-induced y-deflection vectors, ${\underline{\gamma }}_{y}$, are calculated and saved at a sample rate *r* = 200 samples/s. This results in 32 time-series instances of ${\underline{\gamma }}_{y}$ that correspond to the 32 different locations of the cylinder (refer to Section 3.1, for example, responses of ${\underline{\gamma }}_{y}$). Each time-series is then divided into equal portions of time period *T* that are then used to generate the input dataset. The corresponding coordinates of the cylinder in the (x,y) space are also saved in order to generate the target dataset.

When considering the whiskers’ bending responses that correspond to different cylinder positions (presented in Section 3.1), each time-series portion of the deflection vectors of time period *T* is reduced into two 12-element vectors that can capture the reaction of the wake-induced deflection responses to changes in the cylinder’s position. The two vectors are: the deflection mean; $\bar{\underline{\gamma }}$, and the root-mean-square of the deflection fluctuations around the mean; ${\underline{\gamma }}^{\prime }$.

(3) $$\bar{\underline{\gamma }}=\frac{\sum_{i=1}^{N}{\underline{\gamma }}_{y}\left(i\right)}{N},\;N=r\times T$$

(4) $${\underline{\gamma }}^{\prime }=\sqrt{\frac{\sum_{i=1}^{N}{\left({\underline{\gamma }}_{y}\left(i\right)-\bar{\underline{\gamma }}\right)}^{2}}{N}}$$

(5) $$\bar{\underline{\gamma }}={\left[{\bar{\gamma }}_{1}\;{\bar{\gamma }}_{2}\;..\;{\bar{\gamma }}_{12}\right]}^{T},\;{\underline{\gamma }}^{\prime }={\left[\gamma_{1}^{\prime }\;\gamma_{2}^{\prime }\;..\;\gamma_{12}^{\prime }\right]}^{T}$$

**Figure 4:**
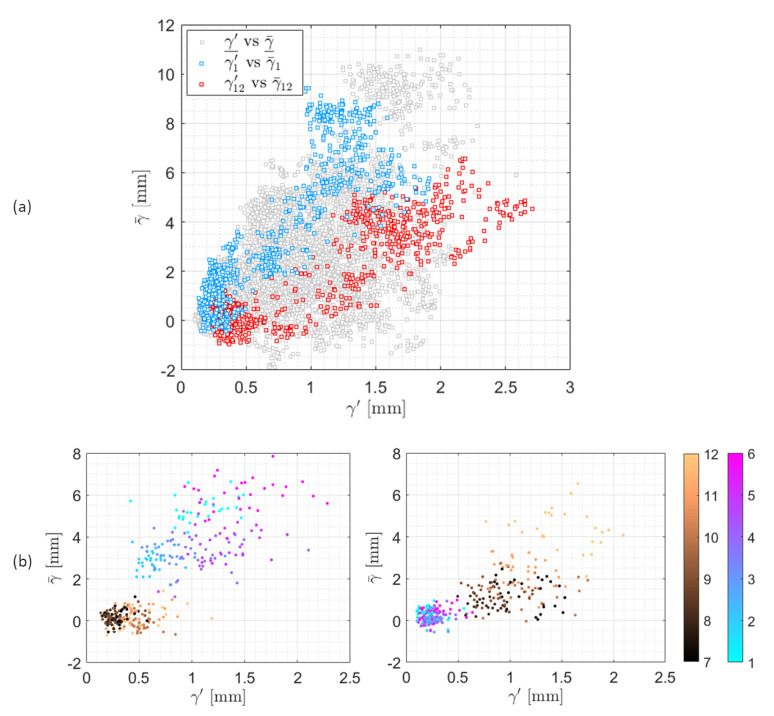
Distribution of the mean and fluctuating deflection samples (inputs to the learning models) for the 12 whiskers in the $\left(\gamma^{\prime },\bar{\gamma }\right)$ space: (**a**) population of the complete input dataset that correspond to all positions of the cylinder. Highlighted in blue and red are the data samples of the whiskers numbered 1 and 12 respectively. (**b**) input samples of two selected recordings that correspond to the cylinder positions (−15,75) and (5,75). The scattered sample points are coloured by their whisker indexes as represented by the colour bar.

The generated data along with the corresponding cylinder positions compose two 24-input-single-output datasets. The input dataset is composed of *Q* samples of the two vectors: $\bar{\underline{\gamma }}$ and ${\underline{\gamma }}^{\prime }$ (each consists of 12 elements that correspond to 12 different whiskers), where the number of samples *Q* is dependent on the choice of the sampling period *T*. The target dataset consists of the separated (*x*, *y*) coordinates of the cylinder positions that correspond to each input sample. Figure 4a shows the population of all the input data in the $I\;R^{2}$ space $\left(\gamma^{\prime },\bar{\gamma }\right)$ during the recordings of 32 positions of the cylinder for T=3 s and Q=772 samples. The input samples of the whiskers numbered 1 and 12 are highlighted in blue and red colours. Figure 4b presents the sample distribution of all whiskers within two different complete recordings that correspond to positions (−15,75) and (5,75) of the cylinder. Different colors in both plots in Figure 4b represent different whiskers. The plots show the diversity of each whisker’s data of the mean and fluctuating tip deflection during a single recording (the same position of the cylinder). The diversity of the deflection data is more significant for the whiskers that are located on the same side as the cylinder is.

### 2.5. Supervised Leaning Models

The wake-induced deflection responses vary differently in response to changes in the *x* and *y* coordinates of the wake-generating cylinder position, as illustrated by Figure 6. Consequently, two separate neural-network models are developed in Matlab, so that each one is responsible for the prediction of a single coordinate of the cylinder position. The input samples are separately prepared to be fed into two 24-input-single-output NNs. Each NN is trained to predict the associated coordinate of the cylinder position (x,y) that corresponds to a given 24-element sample *q* of the input deflection data $\bar{\gamma }$ and $\gamma^{\prime }$ of the 12 whiskers. The selected structures of both NNs as well as their learning algorithms are thoroughly described in Appendices Appendix A and Appendix B, respectively. Different NN structures are tested for the prediction of each coordinate. The selection of the final structures and the optimization of the NN parameters is based on maximizing the prediction accuracies of the models while keeping them as fast and simple as possible. Finally, the FFNN model is used for the prediction of the *x* coordinate, while the TDNN model is used for the prediction of the *y* coordinate with an input-memory (input-layer delay) of four samples.

With a total number of samples of *Q*, the network is trained to optimize the model that associates between the different input-target pairs of the given dataset. The number of samples *Q* is determined by the selection of the sampling period *T*. After setting different values of *T* for different rounds of training for both models, we selected the sampling periods 3 s and 5 s, for the *x*-coordinate model and the *y*-coordinate model respectively.

Two strategies are followed in order to prevent the models from being over-fitted to match the training dataset pairs. The problem of overfitting is a common one when dealing with supervised machine learning and it is thoroughly described in [44,45].

Firstly, the standard 10-fold cross-validation algorithm [46] (developed in Matlab and the code is available with the authors) is performed, as follows: (1) shuffling the training input-target pairs and dividing them into 10 subsets; (2) performing 10 different rounds of the network training iterative algorithm; (3) for each round of training, one of the 10 data folds is used as a validation set while the rest are used for training the network; and, (4) the model accuracy of each round is separately evaluated by calculating the correlation coefficient *R* between the trained model response (output) to the validation set and their given targets. The mean of the correlation coefficients ΣR/10 of the cross-validation rounds is considered to be an approximate representation of the generalized performance of the model.

For additional validation, three extra recordings of the whiskers’ deflection data (with cylinder locations different from those of the original 32 recordings) are processed to come up with approximately Q/13 samples of input-target pairs that would be used as a test dataset. This dataset serves the following purposes: (1) it is used to evaluate the error ranges of the trained-NNs predictions of new cylinder positions in the 2D space (x,y) that were completely excluded from the training itself; (2) the accuracy of the test results is used as a feedback to minimize the number of training iterations of the networks; and, (3) it is lastly merged with the validation subsets of the 10-fold cross-validation to calculate unbiased estimates of the models’ accuracies.

The following parameters are also selected in order to optimize the NN training: the number of NN layers *M* and the number of neurons per each of the *M*-one hidden layers $S_{1,2..M-1}$. The following steps summarize the training procedure and the selection of those parameters:

1. the optimization parameters of the Marquardt–Levenberg Algorithm (MLA) are initialized, as follows: μ=0.001,β=10 (refer to Appendix B for a brief description of the MLA);
2. preliminary values of the two parameters are used, such that the hidden-layer size is set to $\left[S_{1}=15,\;M=2\right]$ for both models;
3. the network training algorithm is performed (as illustrated above) and the overall model accuracy is estimated after the 10-fold cross-validation;
4. the hidden-layer size is then updated by increasing the number of neurons per layer and/or the number of hidden layers and then jumping back to step 3 to restart the training of the networks;
5. after several loops of the above sequence, the hidden-layer size associated with the highest prediction accuracy is selected: [$S_{1}=15,\;S_{2}=16,\;M=3$] for the *x*-coordinate prediction model and [$S_{1}=29,\;S_{2}=13,\;S_{3}=18,\;M=4$] for the *y*-coordinate prediction model.

## 3. Results

### 3.1. Wake-Induced Bending of the Whiskers

The wake-induced deflection vectors, ${\underline{\gamma }}_{x}$ and ${\underline{\gamma }}_{y}$, of the 12 whisker tips in the x and y directions, respectively, are extracted by the processing of the camera output and are defined as:

(6) $${\underline{\gamma }}_{x}={\left[\gamma_{1x}\;\gamma_{2x}\;..\;\gamma_{12x}\right]}^{T},\;{\underline{\gamma }}_{y}={\left[\gamma_{1y}\;\gamma_{2y}\;..\;\gamma_{12y}\right]}^{T}$$

(7) $$\gamma_{nx}=x_{n}-x_{0n},\;\gamma_{ny}=y_{n}-y_{0n},\;n\in \left[1..12\right]$$

where $x_{n}$, $y_{n}$ are the position coordinates of the *n*th whisker tip in the (x,y) space and $x_{0n}$, $y_{0n}$ are the coordinates of the mean position of the *n*th whisker tip due to the free flow *U*_∞_ (without the existence of the cylinder rod).

**Figure 5:**
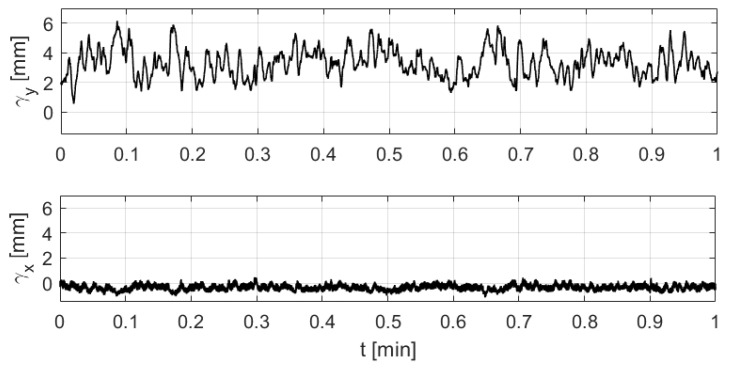
Wake-induced deflection values of a sample whisker in both x (**bottom**) and y (**top**) directions.

As a consequence of the orientation of the 12 whiskers along the lateral axis of the head (the x axis), the y-component ${\underline{\gamma }}_{y}$ of the wake-induced deflection, corresponding to small angles of the whiskers’ vibrations, is expected to be dominant. By examining the wake-induced deflection of a selected whisker in both *x* and *y* directions in the presence of the cylinder (plotted in Figure 5), it is clear that the effect of the cylinder wake on the whisker’s deflection is only noticeable in the *y*-direction.

**Figure 6:**
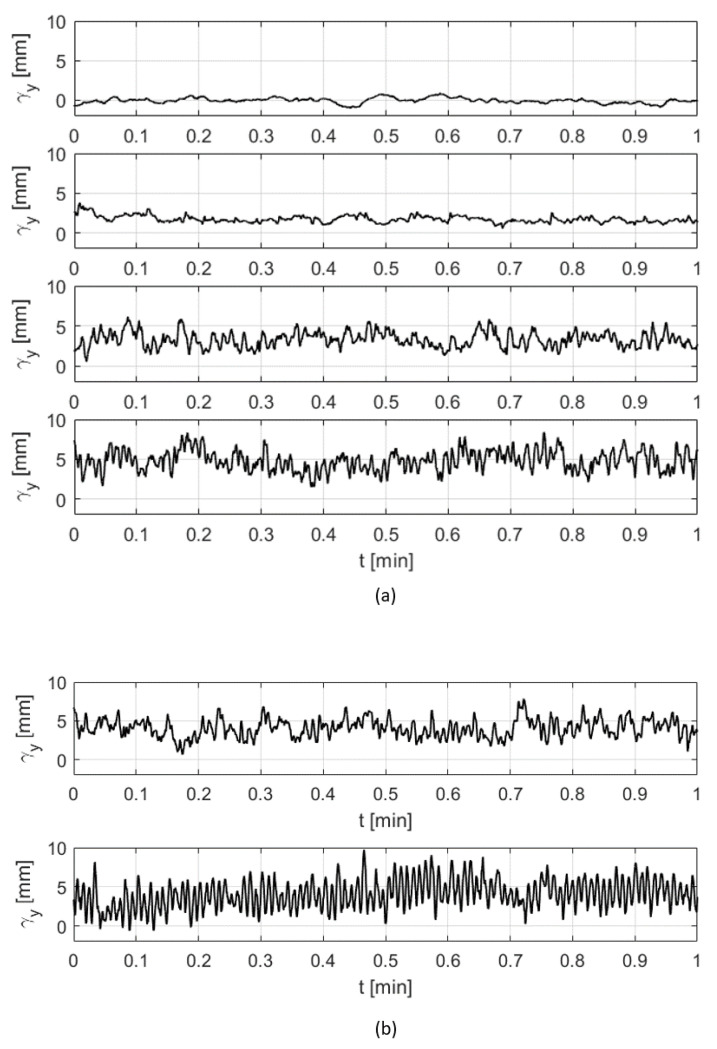
Deflection responses of a selected whisker tip due to the existence of the cylinder at different locations (**a**) variation of the tip’s deflection response due to different *x*-coordinate locations of the cylinder. The curves from top to bottom represent the following cases respectively: no cylinder (reference case), cylinder positions: (−5,75), (0,75), (5,75). (**b**) variation of the tip’s deflection response due to different *y*-coordinate locations of the cylinder. The curves from top to bottom represent the following positions of the cylinder, respectively: (5,85), (5,29).

Now, let us investigate the effect of changing the position of the upstream cylinder on the *y*-deflection curves of the whiskers. In Figure 6a, responses of the deflection in the y-direction are plotted over 1 min. for different locations of the cylinder along the *x*-axis and for a reference case of the free flow with no disturbance. In the reference case, with no cylinder placed upstream, the tip location varies slightly from its nominal position due to the self-induced vibration of the artificial whisker in the presence of the flow and the surface wave of the open channel [28]. After inserting the cylinder, the vortex street in its wake interacts with the whisker and causes a noticeable large-scale variation of both the mean tip deflection and the amplitude of the deflection’s fluctuations around the mean. The deflection response of a whisker that is located on the left side of the head is found to change with the position of the upstream cylinder. Among three different locations of the cylinder, the largest variation of the deflection curve from its reference case (top plot) is found when the cylinder is on the left and centre position with respect to the head axis (i.e., positions (5,75) and (0,75), respectively). This variation gradually shrinks as the cylinder is moved to the right position (−5,75). Similarly, the response of the deflection is found to vary for different locations of the cylinder along the *y*-axis. The fluctuations of the tip deflection curve around the mean (particularly the high-frequency components) get larger as the cylinder moves closer towards the head (from position (5,85) to position (5,29)), as illustrated in Figure 6b for the same selected whisker. Despite that the deflection response is not solely dependent on a single coordinate of the position of the cylinder for a given *Re* and *St* numbers, we believe that each coordinate can be separately deduced by comparing the deflection data of different whiskers that are located on both sides of the head.

### 3.2. Prediction Outcomes of the Learning Models

After the post-processing and reduction of the wake-induced y-deflection data of the 12 whisker tips that correspond to 32 different locations of the upstream cylinder, 24 inputs (two inputs per whisker) are sampled and used to train the leaning models (i.e., each sample is derived from a time-series portion of ${\underline{\gamma }}_{y}$ of a period *T*, as illustrated in the Methods section). Two separate artificial neural networks (NN) are developed to associate between the 24-input samples and each one of the cylinder’s position-coordinates in the (x,y) space. A feed-forward neural network (FFNN) is used for the prediction of the x coordinate, while a time-delay neural network (TDNN) is used for the prediction of the y coordinate. The Methods Section illustrates the selection and parameter optimization of both networks.

**Figure 7:**
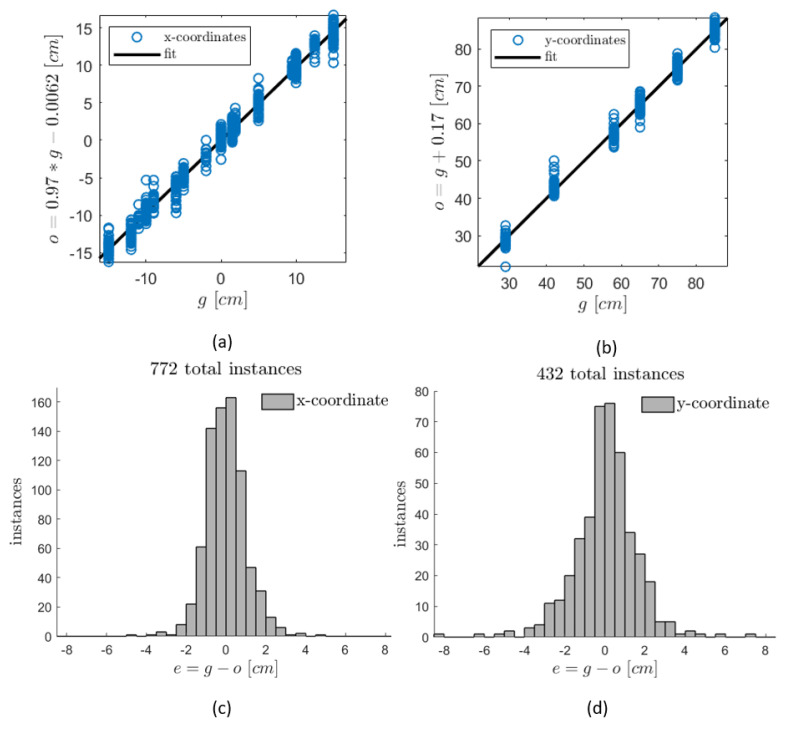
Results of the NNs predictions of (x,y) coordinates of different cylinder locations using the input samples of the training dataset. (**a**,**b**) predicted outputs of (x,y) as a linear fitted function of their labeled targets. (**c**,**d**) histogram plots of the prediction error instances resulted from both models.

The prediction outcomes of the trained NN models of the *x* and *y* coordinates of the cylinder positions are plotted in Figure 7 in the form of regression and error-histogram plots. In Figure 7a,b, the predicted (output) coordinates, represented by the label *o*, are plotted as a linear fitted function of their labeled target values, represented by the label *g*. The slopes of the fitting lines, as well as the correlations between the output/target pairs, show the ability of both models to accurately predict the upstream cylinder position. The error histograms, as plotted in Figure 7c,d, show satisfactory ranges of the prediction deviations from the true target values when considering the small size of the training datasets and the measurement inaccuracies (deviations are represented in the plots by the label *e*). Note that the number of prediction instances is different between the two models due to the choice of different sampling periods *T* for both models while preparing their training datasets. Increasing the size of the training samples has been attempted by overlapping the time-series portions of data that are processed to generate training inputs, described in Equations (3) and (4). However, the overlapping did not have much influence on the resulted error ranges. It is expected that increasing the size of training dataset shall be done by performing more recordings of further positions of the cylinder as well as increasing the recording period to have more portions of *T*-sampled data. The accuracy of the model as a function of the training-dataset size is investigated later in this section.

**Figure 8:**
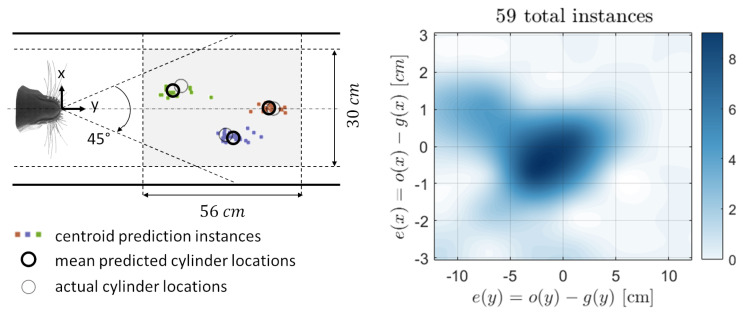
Results of the NNs predictions of (x,y) coordinates of three different cylinder locations using the input samples of the test dataset. For a total of 59 input samples and three different locations of the cylinder, the prediction instances of each location and their mean are represented by different colours (**left**). The two-dimensional (2D) error histogram (algorithm available here [47]) is presented as a colour-contour plot with a total number of 25 bins and a biharmonic interpolant fitting function (**right**).

For further validation of the networks’ training, the resulted models are tested to predict three positions of the upstream cylinder that have not been included in the training stage. The test datasets of both models are acquired from the processing and reduction of the three extra recordings. Figure 8 presents the synchronized models’ prediction results of the test samples. With an average absolute error of about 1 cm and 3 cm for the *x* and *y* models, respectively, around 85% of the resulted predictions lie in a 2 cm×6 cm area around the true value of the cylinder position. However, one can notice that some predictions of the *y*-coordinate have larger deviation amounts from the true value (up to a maximum of 12 cm). This might be due to the relatively small size of the y-model training dataset. It can also be due to the fact that the recordings are not continuous and that the unrealistic discrete changes in the cylinder position are hard to predict for the TDNN model that possesses a dynamic behaviour. Overall, the test results show good agreement with the training validation outcomes in terms of the mean prediction accuracy and the ranges of output error.

By considering the accuracy of the test results, an unbiased estimate of the general accuracy of the NN models can be obtained by merging the test dataset samples with the validation subsets of the 10-fold cross-validation and calculating the correlation coefficient *R* between the model response to the merged input data and their given targets. For output-target paired data that consist of *n* pairs, the coefficient *R* is calculated as

(8) $$R=\frac{\sum_{b=1}^{n}\left(o_{b}-\bar{o}\right)\left(g_{b}-\bar{g}\right)}{\sqrt{\sum_{b=1}^{n}{\left(o_{b}-\bar{o}\right)}^{2}}\sqrt{\sum_{b=1}^{n}{\left(g_{b}-\bar{g}\right)}^{2}}}$$

in which *o* represents the output, *g* represents the target, $\bar{o}$ and $\bar{g}$ are their mean over the given number of pairs, respectively.

Note that the models are still trained with the same training datasets that do not include input samples that correspond to the three test positions of the cylinder. In this case, the mean correlation value at the end of the cross-validation algorithm is found to be 98.68% and 96.15% for the *x*-coordinate model and the *y*-coordinate model, respectively.

### 3.3. Sensitivity of the Predictions to the Size of the Training Dataset

The dependency of the models’ prediction accuracy on the size of their training dataset is studied. For each model, the training is restarted with a different number of input/target pairs fed into the NNs. For each size of the training dataset, the accuracy estimate of the models’ prediction is calculated. Finally, the scores are recorded in Table 1. The accuracies of both models tend to decrease as the sizes of their training datasets get smaller. However, one can notice that the *y*-coordinate model’s accuracy is more sensitive to the training dataset size. Because the TDNN model is used for the *y*-coordinate prediction, it is considering *d*+1 samples of inputs at a given training iteration, where *d* is the model’s memory size (i.e., number of input-layer delayed samples). On the other hand, the *x*-coordinate model is only considering a single sample of the input vector at a given iteration. Therefore, it is expected that the *y*-coordinate prediction can be improved further by increasing the input dataset size. Another reason might be that, although the whiskers’ responses are recorded for 32 different cylinder positions, the y coordinates of these positions vary on only six discrete levels. Although the current performance of the NN models is satisfying, whiskers’ deflection data that correspond to more y-levels of the cylinder locations can be used to reduce the *y* prediction error ranges.

### 3.4. Sensitivity of the Predictions to the Number of Whisker-Pairs Included in the Training

Table 2 shows the effect of varying the number of whisker pairs used in the preparation of the training datasets on the accuracy results of both models. The number of whisker pairs is varied, such that one pair indicates the right and left whiskers that have the same index when the whiskers are ordered according to their position on the *y*-axis (whiskers that have approximately the same y position). At first glance, one can say that the accuracy of the models has a direct relation to the number of whisker pairs involved in the training. The more whiskers included, the more accurate the prediction. It is believed that the flow disturbances, including any noise, do not have the same effect on the deflection responses of different whiskers, because they are located at different locations in the 3D space and due to slight variations in their sensitivities. Therefore, increasing the number of whiskers could be providing the NNs with the ability to filter out the noise in the flow and better decode information about the wake source. It is believed that the *y*-coordinate model is more sensitive to the variation of the number of whisker pairs included in the training due to the same reasons that are detailed in the paragraph above (while considering the accuracy sensitivity to the training-dataset size). Table 2 also compares the two cases when the number of whisker pairs is firstly varied in the front-to-rear direction and then the other way around. The accuracy of the *y*-coordinate model is noticeably sensitive to the location of the whisker pair. When trained with the front whisker pair, the accuracy of the *y*-coordinate model is almost double its value when trained with the rear pair. This can be explained by the fact that the deflection responses of the rear whiskers are not only due to the wake of the cylinder, but could also be due to the wakes of the frontal whiskers that are located in their upstream direction. It is also shown that the effect of varying the whisker-pair location is almost negligible on the accuracy of the *x*-coordinate model. A possible explanation is that the corresponding pair of whiskers from both sides of the head are used. Even being affected by the whisker-induced wakes, the network compares the deflection responses of both sides and can successfully encode the information about the lateral position (*x*-coordinate) of the upstream cylinder.

## 4. Discussion and Conclusions

Previous experiments have demonstrated the ability of the real seal to detect and track its prey while using the information acquired by its facial whiskers about the surrounding water disturbances. In this study, an artificial sensor, inspired by the seal’s whiskers, is developed using machine learning and tested for underwater 2D position detection of a wake-generating body. The sensor consists of an array of optical fibers that are illuminated from one end and inserted through holes from the backside of a 3D printed model of a pinniped’s head and exited from its frontside. The free endings of the fibers act like artificial whiskers that are distributed on both sides of the head. The head, with the artificial whiskers, is mounted inside an open-surface water tunnel that possesses a flow-speed of 30 cm/s. The whiskers are then subjected to the wake that is generated by a cylinder (*Re* ≃ 6000) placed at different upstream locations. A high-speed camera, with a special online optical tracking feature, is used to record the wake-induced vibrations of the whiskers at a frame rate of 200 frames per second. The acquired data are processed and reduced in order to generate the input dataset for the neural networks’ training. With their targets (output labels) being the separated (x,y) coordinates of the different cylinder positions, two neural networks are trained using the Marquardt–Levenberg learning algorithm and the 10-fold cross-validation technique to associate between the input/target pairs.

The measured signal herein is the tip deflection of each optical fiber simultaneously, which is directly proportional to the applied bending moment (Euler–Bernoulli beam theory). Although other alternatives to directly measure the bending of the fibers are known and feasible, this path was initially chosen due to the availability of the optical tracking camera. In our future work, we aim to implement Bragg-gratings in the optical fibers to estimate the bending fluctuations from the corresponding shifts in the reflected Bragg-wavelength. This method, well established in fiber-optical strain or bending sensors, would allow us to capture the signal from inside an underwater vehicle while the fibers’ free ends protrude from the body as in the current application. Another alternative is the implementation of strain gauges to measure the bending strain of the artificial whiskers. Such embedded methodologies would facilitate the integration of the sensor into realistic underwater vehicles.

The trained models can accurately predict the upstream locations of the cylinder that correspond to the training samples of the deflection data as well as the samples of a stranger test dataset, which corresponds to three new cylinder locations. The developed sensor can passively sense the wake and deduce the position of its source with an average absolute error of about 1 cm for the *x*-coordinate prediction and 3 cm for the *y*-coordinate prediction. The reported average error is less than the characteristic parameter, *D*, of wake-generating body and for distances larger than 25-times *D* between the sensor and the body (limited by the size of the water tunnel). The accuracy of the resulted predictions is found to be sensitive to the training dataset size and the number of cylinder locations associated with the recorded deflection data. The accuracy is also found to vary with the number and location of the whisker pairs that are involved in the training process.

The study is limited by the size of the water-tunnel test section and the number of cylinder locations in the performed measurements. Further data collection with more cylinder positions can be performed in the future to minimize the prediction errors of the models. Additionally, as a consequence of the equipment limitation in the water tunnel, the captured recordings of the whisker vibrations is not continuous (i.e., they correspond to discrete changes of the cylinder position). It is expected that, given a continuous variation of the cylinder position in a real-time recording experiment, the NNs can be trained to perform online trajectory-tracking of the upstream cylinder. It is also of interest to test the tracking capability in a larger environment, as we know from previous studies that the wake of a prey can last visible in water for several minutes (more than 3 min. for a small goldfish of a 10 cm body length [13]). The characteristic wake generated by the cylinder in the current implementation of the experiment is comparable to that generated by several fish and cruising submarines. The flow speed used herein is also comparable to the speeds of existing underwater vehicles. Therefore, the responses of the artificial whiskers in such environments are expected not to vary significantly from the current reported ones.

The NN models need to be trained for several scenarios in an otherwise realistic and time-variant environment in order to use the present sensor in underwater-vehicles for tracking applications. The trained network models can then work in real-time with the data acquisition system (i.e., that captures the vibrations of the whiskers and generates the corresponding input data for the models) to continuously update the location of the detected wake’s source. Large-scale water currents are not expected to affect the tracking capability of the neural networks, as their influence would be seen along all the individual whiskers in a coherent manner. In addition, the high-frequency noise due to the self-induced vibrations of the whiskers appear to be identified and possibly suppressed automatically within the NNs, which receive their input data from all whiskers simultaneously. This would help the current tracking models to be trained and used within a noisy environment.

In light of the challenges that face the current techniques of underwater surveillance, the presented sensor demonstrates an alternative methodology of target-tracking that can be utilized in autonomous-underwater-vehicles. The sensor implements a stealthy and passive way of perception that is suitable for use in dark or muddy underwater environments. It possesses a minimum level of environmental impact by featuring a lifelike and safe way of sensing, which facilitates smooth integration with the surrounding marine life.

## Figures and Tables

**Table 1:** Sensitivity of the prediction accuracy (mean[R]) of the (x,y) models to the size of the training dataset.

Size of Training Set of x-Model [Samples]	Mean[R] for Predictions of *x*	Size of Training Set of y-Model [Samples]	Mean[R] for Predictions of *y*
772	0.9868	432	0.9615
708	0.9795	400	0.9231
644	0.9845	368	0.9332
580	0.9757	336	0.8681
516	0.9734	304	0.8517
452	0.9659	272	0.8099
388	0.9789	240	0.7223
324	0.9718	208	0.6166
260	0.9429	176	0.7367
196	0.9459	144	0.6472

**Table 2:** Sensitivity of the prediction accuracy (mean[R]) of the (x,y) models to the number of whisker pairs included in the training stage. The number is varied in the front-to-rear direction of the whiskers (top) and vice versa (bottom).

Number of Whisker Pairs in the Training Set	Mean[R] for Predictions of x	Mean[R] for Predictions of y
6	0.9868	0.9615
5	0.964	0.8843
4	0.9851	0.8686
3	0.9738	0.763
2	0.9512	0.8447
1 (front pair)	0.9536	0.6604
6	0.9868	0.9615
5	0.98	0.9120
4	0.978	0.8766
3	0.9781	0.6595
2	0.9583	0.5928
1 (rear pair)	0.9532	0.3305

**Figure A1:**
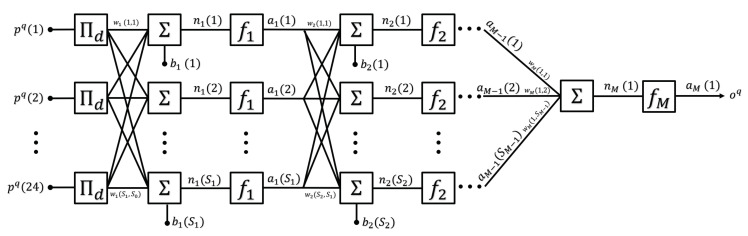
Structure of the *M*-layer feedforward neural networks used for the prediction of the *x* and *y* coordinates of the cylinder position.

## Appendix A. Structures of the Neural-Network Models

The structures of both the *x*-coordinate and *y*-coordinate networks can be described by the schematic in Figure A1. The diagram represents a feedforward network structure with *M* layers; (*M* − 1) hidden layers and one output layer, and an optional input-memory function $\Pi_{d}$.

The network input $\underline{n}$ to the layer k+1 is a linear function of the output $\underline{a}$ of its preceding layer *k*. In the following analysis until the end of this section, the sample indexes and the layer indexes are represented by superscripts and subscripts respectively.

(A1) $${\underline{n}}_{k+1}=W_{k+1}{\underline{a}}_{k}+{\underline{b}}_{k+1},\;W_{k+1}{\underline{a}}_{k}=\sum_{j=1}^{S_{k}}w_{k+1}\left(i,j\right)a_{k}\left(j\right)$$

where $S_{k}$ is the size of the layer *k* (i.e., number of elements of vector ${\underline{a}}_{k}$), k∈{0,1..M−1} is the layer index, ${\underline{b}}_{k+1}$ is the biases vector and $W_{k+1}$ is the weights matrix of layer k+1.

The network output $\underline{a}$ of the layer k+1 is

(A2) $${\underline{a}}_{k+1}=f_{k+1}\left({\underline{n}}_{k+1}\right),\;f_{1,2,.M-1}\left(n\right)=\frac{2}{1+e^{-2n}}-1,\;f_{M}\left(n\right)=n$$

${\underline{a}}^{0}$ is connected to the input vector $\underline{p}$, that represent the deflection data of the whisker tips, via the input-memory function $\Pi_{d}$ of which d=0 for *x*-coordinate prediction (direct connection) and d=4 for *y*-coordinate prediction.

(A3) $$\Pi_{0}\left(p^{q}\right)=p^{q},\;\Pi_{\left\{d|d\in N\right\}}\left(p^{q}\right)={\left[p^{q}\;..\;p^{q-d}\right]}^{T},\;$$

(A4) $${\underline{a}}_{0}=\Pi_{d}\left(\underline{p}\right)$$

$a_{M}$ is connected to the network output *o* and the error *e* is defined as the difference between the target label *g*, that represents one of the two coordinates of the cylinder position (x,y), and the output of the network *o*.

(A5) $$a_{M}=o^{q},\;e^{q}=g^{q}-o^{q}$$

where *q* is the sample index and *d* is the memory size of successive input samples. The addition of a d-sample input-memory provides the model with a dynamic response which could add a noticeable gain to its efficiency. Such networks with a finite-time input-memory are known as time-delay neural networks (TDNN) and have been reported to be particularly efficient in speech-recognition, property-prediction and automatic-control applications [48,49,50].

## Appendix B. Learning Algorithm

The goal of the NN is to map each sample of its input vector to its corresponding given output label [51]. The performance of each network model is evaluated by the cost function *C* which is defined as the mean squared error (MSE) of all output-target pairs.

(A6) $$C=\frac{1}{Q}\sum_{q=1}^{Q}{\left(e^{q}\right)}^{2}$$

in which *Q* is the total number of samples.

In order to minimize the cost function, the Marquardt-Levenberg algorithm [52] (i.e., an optimization of the of the steepest descent method [53] used in the standard backpropagation NN learning [51]) is used to update the weights and biases of the NNs. It updates the network weights and biases in each iteration as follows [54]:

(A7) $$\Delta \underline{z}={\left[J^{T}J+\mu I\right]}^{-1}\underline{\nabla }C,\;J\left(i,j\right)=\frac{\partial e(i)}{\partial z(j)}$$

where $\underline{z}$ is the parameter vector which contains all weights and biases, $\underline{e}$ is the error vector; $\underline{e}={\left[e^{1}...e^{Q}\right]}^{T}$ and *J* is the Jacobian matrix of the network error. The term ${\left[J^{T}J+\mu I\right]}^{-1}$ substitutes the learning rate of the original steepest decent algorithm. The separate term $J^{T}J$ is the Gauss-Newton approximation of the Hessian matrix of the cost function; H(C). The parameter μ is initialized with a small value and is multiplied by a factor β each time the update of $\underline{z}$ results in an increase in the value of *C*. As μ gets larger, the algorithm approaches a steepest descent algorithm with a learning rate of 1/μ.
